# Factors affecting medication adherence: patient perspectives from five veterans affairs facilities

**DOI:** 10.1186/s12913-014-0533-1

**Published:** 2014-11-13

**Authors:** Clarissa Hsu, Jaclyn M Lemon, Edwin S Wong, Elizabeth Carson-Cheng, Mark Perkins, Margaret S Nordstrom, Chuan-Fen Liu, Carol Sprague, Christopher L Bryson

**Affiliations:** Center for Community Health and Evaluation, Group Health Research Institute, 1730 Minor Ave, Suite 1600, Seattle, WA 98101-1448 USA; VA Puget Sound Health Care System, Northwest Center for Outcomes Research in Older Adults, 1660 South Columbian Way, Seattle, WA 98108 USA; Department of Medicine, University of Washington, Box 356420, , 1959 NE Pacific Street, Seattle, WA 98195 USA; Department of Health Services, School of Public Health and Community Medicine, University of Washington, 1959 NE Pacific St. Magnuson Health Sciences Center, Box 357660, Seattle, WA 98195 USA; Portland VA Medical Center, 3710 SW U.S. Veterans Hospital Road, Portland, OR 97239 USA; Oregon Health and Science University, 3181 S.W. Sam Jackson Park Rd., Portland, OR 97239-3098 USA

**Keywords:** Diabetes, Medication adherence, Chronic disease management, Patient self-management, Delivery of care, Veterans, Health systems research, Pharmacy services research, Patient-oriented research

## Abstract

**Background:**

In the United States, more than 25 million people have diabetes. Medication adherence is known to be important for disease control. However, factors that consistently predict medication adherence are unclear and the literature lacks patient perspectives on how health care systems affect adherence to oral hypoglycemic agents (OHAs). This study explored facilitators and barriers to OHA adherence by obtaining the perspectives of Veterans Affairs (VA) patients with OHA prescriptions.

**Methods:**

A total of 45 patients participated in 12 focus groups that explored a wide range of issues that might affect medication adherence. Participants were patients at clinics in Seattle, Washington; San Antonio, Texas; Portland, Oregon; Salem, Oregon, and Warrenton, Oregon.

**Results:**

Key system-level facilitators of OHA adherence included good overall pharmacy service and several specific mechanisms for ordering and delivering medications (automated phone refill service, Web-based prescription ordering), as well as providing pillboxes and printed lists of current medications to patients. Barriers mirrored many of the facilitators. Poor pharmacy service quality and difficulty coordinating multiple prescriptions emerged as key barriers.

**Conclusions:**

VA patient focus groups provided insights on how care delivery systems can encourage diabetes medication adherence by minimizing the barriers and enhancing the facilitators at both the patient and system levels. Major system-level factors that facilitated adherence were overall pharmacy service quality, availability of multiple systems for reordering medications, having a person to call when questions arose, counseling about the importance of adherence and providing tools such as pillboxes and updated medication lists.

**Electronic supplementary material:**

The online version of this article (doi:10.1186/s12913-014-0533-1) contains supplementary material, which is available to authorized users.

## Background

Diabetes is a serious and increasing US health concern that affects over 25 million people [1]. Within the Veterans Affairs (VA) health care system, diabetes is even more common, affecting 25% of VA patients in 2010 [1]. Successful management of this complex disease requires a long-term and multipronged approach. As most diabetes patients are unable to achieve sufficient glycemic control through diet, exercise and weight loss alone, pharmacologic therapy is a key treatment component [2]. Of US adult patients with type 2 diabetes, 72% take at least one oral hypoglycemic agent (OHA) [1]. However, poor adherence to medication is a persistent problem across many diseases, especially chronic conditions like diabetes [3]. Research also suggests that adherence is highly variable among people with diabetes, indicating that patients take only 7% to 64% of their anti-diabetic drug doses [4]. Poor adherence to OHAs is linked to intermediate and long-term health outcomes such as higher hemoglobin A1c levels [5,6], increased symptoms, and poorer physical and mental functioning [7]. Nonadherence to diabetes medications is also associated with increased health care utilization and higher mortality [5,6].

Patient-level factors predictive of medication adherence are well established and include age, sex, race/ethnicity, education, and income or socioeconomic status [8-10]. In addition, for patients managing diabetes, qualitative studies have identified a number of common patient challenges and/or needs are: 1) information needs [11-17]; 2) personal experiences with illness, medications, and complications [12,13,17-20]; 3) social support, including the patient-physician relationship [12,15,18,19]; 4) lifestyles and routines [19,21,22]; 5) costs [16,17,19,20]; and 6) psychosocial factors [13,17,19,20]. Only a few qualitative studies have specifically focused on diabetes medication adherence; their findings are generally consistent with the larger body of qualitative work on overall diabetes self-management [21-25].

Although individual studies indicate that patient level-factors are important to medication adherence, meta-analyses suggest that no single, robust patient-level factor predicts medication adherence [26]. Additionally, many patient-level factors such as income or socioeconomic status are largely immune to modification by health care systems. From the perspective of health systems such as VA, understanding organizational factors associated with medication adherence is important to improve delivery of care. Understanding organizational correlates of adherence is also important given the American Diabetes Association recommendation that patients with diabetes see a health provider every three months [27]. The literature suggests that provider-level factors such as the patient-physician relationship and communication, [28,29] provider attitudes toward diabetes [30,31], and provider knowledge and skills related to diabetes treatment [30,32] have the potential to influence adherence behavior. However, other potential organizational/health system correlates of adherence have received far less attention.

We found no studies that specifically asked patients what aspects of their health care system affect their adherence to OHAs. While understanding patient-level factors is critical to ensuring that system-level interventions are designed to meet patient needs, we must also understand patient perspectives on how health care systems either facilitate or hinder medication adherence. Given the frequent interaction diabetes patients have with health care providers, facilities, and the overall delivery system, patient perspectives are important for understanding how systems can better support the needs of patients dealing with chronic diseases such as diabetes. Using patient focus groups, our study examined the experiences and perspectives of VA patients who take OHAs to elicit organizational facilitators and barriers that contribute to their medication adherence. Since many of the study participants took medications for a variety of conditions, our findings may be more broadly applicable to patients with multiple chronic diseases as well as those on OHAs.

## Methods

### Focus groups

This research was part of a larger study of VA primary care clinics to assess variation in OHA medication adherence and identify organizational factors associated with better medication adherence. To obtain patients’ perspectives, we conducted focus groups exploring issues that might affect OHA adherence. Focus groups were chosen in order to increase the diversity of opinions we were able to gather. The focus group approach also allowed us to explore if there were common issues raised at particular sites and by the low adherence versus high adherence patients. The focus group interview guide included topics such as patient views on adherence to OHA medication regimens, challenges to taking medications as prescribed, refill methods, VA system barriers and facilitators, attitudes toward self-care, and openness to using technology for assistance with medication adherence (see Additional file 1 for full guide).

### Study population and recruitment

Potential participants were identified from a national sample for the main study of 444,418 type 2 diabetes patients in 559 VA primary care clinics. We identified 11 potential clinics based on diversity of clinic-level adherence rates and geography [33]. Five clinics in Seattle, Washington; San Antonio, Texas; Portland, Oregon; Salem, Oregon; and Warrenton, Oregon agreed to participate and met the requirements for accredited human research protections program oversight. The goal was to recruit 4 groups of 6-8 participants at each clinic. Participants were defined as either adherent or non-adherent using a validated medication possession ratio [34]. The ratio reflects the proportion of days during the quarter (in this study, the first quarter of fiscal year 2007) that the patient had all medications in their diabetes regimen. Medication possession was ascertained using pharmacy refill records in VA administrative databases covering all VA facilities nationwide. Patients were defined as adherent if they had medication available for at least 80% of the time period, a cutoff commonly used in studies of refill-based adherence measures [33]. After excluding patients who died before September 2009 or no longer resided in the clinic’s state, random samples of 50 adherent and 50 nonadherent participants were drawn from each clinic. If a clinic had fewer than 50 potential participants, all eligible patients were invited. In total, 497 potential focus group participants were identified.

Potential volunteer participants were mailed a letter with an information statement or informed consent form (depending on the local Institutional Review Board requirements), and a prepaid “opt-out” postcard. Patients who did not opt out were called about participating. Of 497 potential participants who were mailed letters, 71 agreed to participate; of these, 25 did not appear at their scheduled focus group session (despite multiple reminder calls), and 1 withdrew midsession, for 45 total participants in 12 groups across 5 clinics. Groups contained only adherent or non-adherent patients with no mixed groups. The number of participants per clinic ranged from 5 to 15. Individual group session sizes were 1 to 9 participants. For two sessions, only one of the scheduled participants showed up. In these cases, a semi-structured interview using the focus group guide was conducted and transcripts were included in the analysis. Patients received $25 for participation.

All participants reviewed and signed a written informed consent form before participating which discussed issues of confidentiality; including that fact there their names would not be associated with any quotes used. The methods and procedures for this study were reviewed by the Institutional Review Boards at each of the clinic sites where focus groups were conducted:1) Veteran Affairs Puget Sound Health Care System, Office of Research & Development, Veteran Affairs Puget Sound Human Research Protection Program, 2) South Texas Veterans Health Care System Institutional Review Board, 3) Portland Veteran Affairs Medical Center Institutional Review Board. All protocols were also reviewed by the institutions where key personnel were located: 1) Group Health Research Institute’s Institutional Review Board and 2) the University of Washington’s Human Subjects Division. Most of the staff working on the focus groups were professional research staff and did not occupy dual roles in research and care delivery. The one exception was the Principal Investigator who also provides clinical care, however none of his patients were involved in the focus groups.

### Data analysis

**Figure 1:**
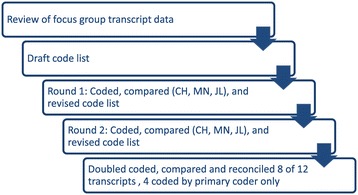
**Process for coding transcript data.**

Audio recordings were transcribed and deidentified by a study team member (JL) who attended all focus groups. Each participant had a unique ID number. A coding scheme was developed based on issues that emerged while reviewing transcript content and *a priori* topics of interest. Three coders (CH, MN and JL) coded one of the transcripts using the code list, then discussed and reconciled differences. After revising the code list and definitions, a second transcript was coded by all coders followed by another round of discussion and revision. After coding the second transcript, the team determined that agreement around the code list and definitions was sufficient to code the remaining transcripts. A single team member coded all 12 transcripts (CH), with 8 also coded by a second team member (MN or JL). The primary and secondary coders reviewed and reconciled differences. Figure 1 summarizes this process. Atlas.ti software assisted with data management [35,36]. Data were sorted by high- and low-adherence groups and reviewed by the primary coder (CH) to ensure code accuracy and to identify important subthemes. A coding memo summarizing findings and example quotations was developed and reviewed by the research team for face validity, appropriate breadth of analysis, and themes for further exploration.

Patient-identified facilitators and barriers included system-level and patient-level factors. This study defined system-level factors as all issues linked to the organization and distribution of health care, including provider behavior and patient interaction with health systems. Patient-level factors were defined as activities or issues over which the patient had direct control or experienced daily such as lifestyle behaviors, organizational habits, and emotional response. In many cases, themes that patients identified as patient-level factors were linked to system-level factors. This study focused only on system-level factors, as patient-level factors have been studied and our findings were consistent with prior research [33].

## Results and discussion

### Participant characteristics

Of the 12 focus groups at 5 sites, 7 contained high-adherence patients. Participant ages were 50 to 89 years, with an average of 69 years across all sites (Table 1). Participants were predominantly male with an average of 2–3 primary care visits in the index year. Nearly three-quarters were eligible for free care in the VA system.

**Table 1:** **Focus group participant characteristics**

**Characteristic**	**All patients**	**Patient focus group**											
		**A1** ^**†**^	**A2**	**A3**	**B4**	**B5**	**C6**	**C7**	**D8**	**D9**	**E10**	**E11**	**E12**
**Location**	**5 sites**	**A**	**A**	**A**	**B**	**B**	**C**	**C**	**D**	**D**	**E**	**E**	**E**
N	45	4	3	3	1	4	9	6	4	3	5	2	1
Age mean in years (SD), [range]	69.3 (8.7) [50-89]	69.3 (6.2) [64-76]	70.0 (17.1) [56-89]	66.7 (11.0) [58-79]	68	70.5 (4.4) [64-74]	69.3 (9.8) [58-78]	69.8 (11.1) [50-82]	72.0 (11.8) [64-89]	69.7 (5.1) [64-74]	69.6 (8.8) [62-83]	63.5 (0.7) [63-64]	66.0
Female, N (%)	3 (6.7)	0	0	1 (33.3)	0	0	1 (11.1)	1 (16.7)	0	0	0	0	0
Married, N (%)	29 (64.0)	3(75)	3 (100)	3 (100)	1 (100)	3 (75)	5 (55.6)	3 (50)	2 (50)	0 (0)	5 (100)	1 (50)	0
White race, N (%)	32 (71.1)	3 (75)	0	2 (66.7)	1 (100)	2 (50)	7 (77.8)	5 (83.3)	3 (75)	2 (66.7)	5 (100)	1 (50)	1 (100)
VA primary care visits, mean (SD) [range]	3.1 (2.3) [1-16]	3.3 (0.5) [3,4]	1.7 (0.6) [1,2]	3.7 (1.2) [3-5]	3	6.8 (6.2) [3-16]	1.7 (0.7) [1-3]	3.1 (1.7) [1-6]	2.2 (1.0) [1-3]	2.7 (0.6) [2,3]	4.0 (0.7) [3-5]	1.5 (0.7) [1,2]	5
Distance to VA in 2010 in miles, mean (SD), [range]	14.9 (12.0) [0^*^-43.5]	10.8 (6.8) [3.9-19.9]	12.0 (8.9) [2.7-20.5]	13.6 (2.0) [11.8-15.8]	6.9	16.0 (12.2) [3.9-29.9]	13.4 (11.2) [0^*^-29.0]	11.8 (9.2) [0^*^-22.9]	6.8 (3.7) [2.8-11.1]	12.8 (13.9) [3.9-28.8]	29.6 (19.2) [5.5-43.5]	18.1 (8.8) [11.8-24.3]	39.5
Free care in VA, N (%)	33 (73.3)	3 (75)	3 (100)	3 (100)	1 (100)	4 (100)	6 (66.7)	4 (66.7)	2 (50)	3 (100)	2 (40)	2 (100)	0
High school graduate, % of population in subject’s county (SD), [range]	84.0 (4.7)[76.9-90.3]	90.0 (0.6) [89.2-90.3]	89.9 (0.6) [89.2-90.3]	89.9 (0.6) [89.2-90.3]	76.9	78.7 (3.5) [76.9-83.9]	81.0 (3.2) [79.3-88.9]	80.5 (1.8) [79.3-82.8]	86.4 (1.7) [85.6-88.9]	87.8 (1.9) [85.6-88.9]	84.7 (0.8) [84.1-85.6]	82.3 (4.7) [78.9-85.6]	84.1

^†^The letters indicate a particular site while the numbers provider a unique identifier for each focus group.

^*^Subject has the same zip code as VA facility’s zip code.

Participants in all groups reported comorbidities including dementia, post-traumatic stress disorder, arthritis, cardiac disease, hypertension, glaucoma, chronic obstructive pulmonary disease, and cancer. Although participants were not specifically asked about the number of medications they were taking, 18 mentioned being on complex medication regimens in addition to OHAs, with 7 reporting 10 or more different medications and 2 reporting more than 27 different medications. Comorbid conditions and a high level of medication usage affected OHA adherence in several ways including: memory loss that made it difficult to remember to take medications, negative side effects from drug interactions, and difficulty in managing multiple medications with different administration instructions and refill intervals.

*And I even get to the point that’s I don’t even remember what the pills are that I’m supposed to have, you know. So, I just have to take all my pills up there with me, and there’s like 26 of them, and say, “I need refills.” And then they have to go through every single prescription to see when the refills are ready to be done…(Group C7)*

### System-level facilitators of OHA adherence

Focus group participants discussed a variety of system-level factors that they felt helped them consistently take their diabetes medication. We grouped factors into several general categories: 1) pharmacy-related services that facilitated the medication ordering; 2) provider and staff communication with patients about their medications; and 3) assisting patients in establishing routines to help them take and refill their medications.

Pharmacy-related facilitators included good overall pharmacy service; convenience of automated phone and Web-based refill tools; and an option to mail medications to the patient’s home. Positive comments about overall pharmacy service were not common and were mentioned at only three of the five clinics.

*I’ve had nothing but good response out of [Pharmacy]. When I get low on my pills, I call and I do the automatic order online, and I’ve never had a problem. And even when…they can’t refill it, they get ahold of the physician here. And obviously she signs off “yes” and still wants to prescribe that. And I’m very grateful for that. I don’t have to worry about it and I know it’s going to come, as long as I order them and give them enough time, they tell me 7-10 days and it’s here in 7-10 days. So I have no complaints. (Group C7)*

Patients described a wide variety of helpful system-level tools for ordering refills. These included phone and Web-based prescription-ordering options; information on the medication bottle (refill phone number, number of refills and date); automatic refill systems that require no patient action to trigger the refill; paper mail-in slips included with each prescription order; and having clinic staff remind them about refills during appointments.

A second category of facilitators related to the accessibility and quality of communication with staff and providers. In particular, patients’ ability to reach staff by phone or email about general medication questions or needs was important to medication regime adherence including OHAs. Patients most often mentioned calling a clinic nurse, but occasionally, their doctor or pharmacist filled this role. This facilitator was spontaneously mentioned in all but 2 of the 12 groups.

*I don’t have any problem ordering the medications when I want to order them. Or I lose them, what I do is I call the nurse. They have a day nurse and a night nurse, another day nurse and sometimes they’ll say, “Ok, what’s the medication?” And they’ll say, “Ok, go to the pharmacist.” And just get a small amount of it, until you go and see your doctor, or primary care. (Group B5)*

Another related facilitator was communication with providers or other members of the patient’s care team around the need for medications and the development of an informed and realistic medication routine. Respondents seemed to indicate that they took medication advice from their care team members more seriously than comments from pharmacists.

*And when I first started, I had a schedule that I set up that I’d mark off, so I would get in the habit. And you have a problem when you first start out things. So what I did was sat down with [Dr. A] and I had him explain what you mean for each one. And he explained when I should be taking them, and what would be the best time. And so that I put on my chart and that’s when I take my pills. Which I take my pills throughout the whole day, so there’s different times that I take pills. But sitting down and having him explain what he wanted, because most the time what comes on that bottle comes from the pharmacy. That’s what they know. But, he has his own ideas, and that was a big thing that helped in that part. (Group E10)*

The third category of facilitators helped patients establish personal systems and routines for taking and refilling OHA and other medications. Although patients talked about these factors on a more personal level, they covered issues that could be addressed at a system-level. Numerous patients mentioned the importance of a pillbox or a physical organizing system such as small containers, bottles or zip-top plastic bags for maintaining their medication routine. Another frequently mentioned facilitator was a consistent daily routine; generally, a daily event was used to trigger the taking of medications such as meals, exercise, or television. Some people reinforced routines with an alarm reminder such as the timer on their cell phone. Finally, a few patients used a written list of medications and when they should be taken. Based on respondent comments, these reminder systems not only facilitated daily medication adherence, but also helped patients remember refills. Several respondents noted that setting up the pillbox at the beginning of the week triggered them to order meds before they ran out.

*I fill my canister on Sunday evenings. So at that time I’m looking at each vial of medication to determine how much I have left. So when I’m down to a week’s left in a container, I’ll call it in. (Group B5)*

High adherers mentioned combining daily routines with pill organization more often than low-adherent respondents. Also, respondents in the high-adherence groups mentioned having pillboxes and daily routines twice as often as the low-adherence groups and gave more detailed descriptions of systems and routines. Clinics that provided pillboxes, updated medication lists at routine appointments, and counseling on the importance of OHA medication adherence and routines appeared to support routine patient-level behaviors that ensured adherence.

### System-level barriers to OHA Adherence

Respondents reported a number of system-level barriers to OHA medication adherence. These barriers were primarily in three categories: 1) concerns about the quality of pharmacy services; 2) communication issues including communication breakdowns between care teams and pharmacy; and 3) lack of education and support around specific medication regimes.

The most frequent category of medication adherence barriers was pharmacy-related factors, focusing on poor overall pharmacy service. Items reflecting poor pharmacy service included insufficient staffing, poor quality control, and lack of a customer-service orientation. More specific pharmacy-related barriers were also mentioned and included excessive wait times, both in the clinic and on the phone; inability to reach someone to resolve prescription problems; problems with ordering and/or delivery services; and dissatisfaction with formulary.

*If I had an appointment and I came out here let’s say, 11 o’clock in the morning - had an appointment, had to go down and get some medicine because it was a fresh prescription, new prescription. And I’ve been out here for 5 hours. (Group D9)*

*But here a couple months ago, the (pharmacy) down here - messed up somehow. I don’t know who did it or how they did it or whatever, but I kept calling them and calling them for 6 weeks, 7 weeks. … I couldn’t get ahold of a person. (Group E11)*

Another category of barriers related to communication and the ability of different departments to coordinate medication logistics. Respondents reported communication breakdowns between departments, the most commonly mentioned were between providers and pharmacy. Providers were generally perceived as advocating for patients, but their communications did not always achieve the desired results at the pharmacy.

*I think it’s a doctor-pharmacy problem, because I see my physician every year, once a year, and sometimes more frequently than that, and there’re still mix ups and cancellations. You know, I talked to my doctors about that, now with 2 of them, and from both of them at completely different offices are saying, they fight with VA pharmacy all the time to no avail. And now they’re even saying, “If it has to do with prescriptions, go talk to the pharmacy because I don’t get any different treatment than you do.” (Group C6)*

Another related but separate issue was the lack of alignment in prescription refill timing. Patients with several types of medications had to order different medications at different times, requiring frequent refill orders. Several patients reported that providers cancelled and reordered medications to increase alignment, however even this action did not always resolve the problem.

*I’m taking enough medications though, that almost all of them come in varying units. The metformin comes in 180 pills and luckily the glyburide come in 180 pills. But some of the other pills I take are 30-day supply, 60-day supply, and so even though they’re maintenance and I’ve been taking them forever… it would be really nice if all my pills came with the same supply… and I have forgotten to order them, I thought I had more of that, or I thought I had more of this. (Group A3)*

The final communication-related barrier to OHA adherence was lack of education about medications and/or difficulties managing a complex medication regimen. Five of the 12 groups mentioned a lack of understanding about when to take medications and what types of modifications could be safely made to medication routines. Patients wanted to streamline their medication regimen as much as possible while maintaining safety and effectiveness. Some respondents rejected complicated instructions in favor of a medication schedule they could achieve.

*I take pills that I take before I go to bed because I got one pill that’s supposed to help me sleep. It doesn’t too much. So I take me Metformin every day in the evening at bed time. And then when I get up I take my medicine, about 12 hour difference in between. And I also got another drop I got to take at bedtime. I do all my medicating then. I don’t know if that’s good or bad. (Group E10)*

Related to concerns about education on “doable” medication regimes, participants talked about lifestyle factors that affected adherence. In some cases, the amount of system-level assistance and coaching patients had received to help them modify or accommodate personal barriers was unclear.

Three of the 12 groups mentioned copayments and cost as a barrier. However, respondents did not directly associate cost with not getting medications and medication adherence. A few respondents stated that copayments were difficult to manage on a fixed income; however none stated that they did not reorder medications because of lack of funds or cost.

## Discussion

The findings from our focus groups with VA patients who took OHAs provide patient perspectives on how health care delivery systems facilitate or create barriers to obtaining and taking medications to control diabetes. System-level facilitators included good overall pharmacy service and several specific mechanisms for ordering and delivering medications (automated phone refill service, Web-based prescription ordering). Several important facilitators were linked to communication between the patient and the system including the ability to contact someone with questions and consult with providers regarding rationales for medication choices and problem-solving around daily medication regimes.

Barriers mirrored many of the facilitators with the importance of pharmacy service quality emerging as central. Another important barrier was a lack of alignment among prescriptions, which made it difficult for patients on multiple medications to track and order refills. Insufficient communication and education also emerged as a barrier.

This is one of the first studies to look specifically at system-level facilitators and barriers of OHA adherence from patients’ perspectives. However, many of the themes that emerged were consistent with patient-level correlates of adherence mentioned in the literature such as supporting patients in developing a medication routine and providing clear and consistent communication and education about the reasons for particular medications and schedules. New insights that emerged from this study include the importance of efficient and effective pharmacy systems and the need to align medication schedules to facilitate daily administration and refills. An efficient and effective pharmacy clearly emerged as a central system-level facilitator, with numerous specific factors critical to patient adherence including order and delivery services, wait times, quality control, and being able to reach someone with questions or concerns. Our findings on the efficiency of the VA pharmacy as a facilitator of adherence are consistent with a prior study showing that patients in clinics where the pharmacy is not perceived as a bottleneck in the flow of care exhibit the highest rates of OHA adherence [37].

Innovations in health care information technology and customized medication blister packs could address the challenges identified in this study. Currently, VA is adopting a new model of health delivery by providing care through Patient Care Aligned Teams (PACT), based upon the Patient-centered Medical Home model [38]. Pharmacists are increasingly being integrated into primary care teams that are the central component of PACT. Having a member of the patient’s care team assess the patient’s understanding and beliefs about their diabetes medication, and helping them find tools to integrate OHAs into their lifestyle and self-care routines could address the gaps in care discussed by the focus groups in our study.

### Limitations

While generating important insights, this study also has limitations. First, the VA is a large, integrated health care system with unique advantages and challenges to providing health care. Therefore, not all insights will translate to other settings. Also, the study respondents represented a specific demographic group—older men. However, our findings were consistent with the general literature on diabetes management and medication adherence. OHA adherence was measured for the first quarter of fiscal year 2007 and correlated strongly with annual OHA adherence. Measuring adherence closer to the focus group sessions was not practical because of data lags and human-subject review delays. Since site recruitment was lower than anticipated, we did not explore how site-specific systems influence adherence. We also had challenges recruiting individuals for the low-adherence groups, so their perspectives might not be represented as well as high-adherence patients.

## Conclusions

Adherence to an OHA medication regimen is critical to many people with diabetes. With diabetes predicted to affect 52% of Americans by 2020, guiding patients in effectively managing this chronic condition is increasingly important for preventive care [39]. OHAs and similar medications can minimize complications and prevent or delay disease progression. However, research shows that many patients have difficulty adhering to OHA medication regimens [5,8]. This study draws insights from focus groups with VA patients to understand how care delivery systems can design system-level and patient-level processes and services to minimize barriers and enhance facilitators to OHA adherence. Factors that encouraged medication adherence tended to simplify, routinize, and standardize the processes of taking and refilling medicines in general. Other factors ensured that trusted members of the patient’s care team answered medication-related questions. We hope that this research will help spur system-level innovation and experimentation that improves medication adherence and increases the quality and efficiency of care for diabetic patients. Furthermore, many of the insights discussed here could apply to other chronic conditions that require adherence to a complex daily medication regimen.

## Additional file

Additional file 1
**Focus Group Question Guide.**

## Abbreviations

OHA: Oral hypoglycemic agents
VA: Veterans Affairs
