# Supplementary material for: Factors affecting medication adherence: patient perspectives from five veterans affairs facilities
Source: BMC Health Serv Res. 2014 Nov 13;14:533. doi: 10.1186/s12913-014-0533-1 (PMC4239388; doi:10.1186/s12913-014-0533-1)
Supplement: Additional file 1 — Focus Group Question Guide. [file 12913_2014_533_MOESM1_ESM.doc]

***Factors Affecting Medication Adherence: Patient Perspectives from Five Veterans Affairs Facilities***

**Focus Group Guide**

1. I’d like to start by having each of you **introduce yourself**. For your introduction if you could tell us 1) **your name**, 2) your current or former **profession**, 3) **how long** you have had diabetes and 4) **one other fact** about you (can be anything, like a hobby, how many grandchildren you have, your favorite TV show, etc).
2. How do you feel like you are you doing taking your medication for your diabetes?
   1. What challenges are you having?
   2. What are some of the things you do to help you take your medications?
3. How do you normally refill your prescription for your diabetes medication?

Probes:

- …Phone
- …Mail
- …Internet
- …In-person
  - …At the pharmacy
  - …At your doctor’s clinic
  - …At the ER or urgent care center
- Have you ever found it difficult to (ask for specific stories)
  - - - …get your prescriptions refilled? …Including when your refill runs out?

1. What are the things that the VA does that make it hard for you to take the medications you need to manage your diabetes?

Probes:

- - - Can you tell us about a specific time you had that problem? When did it happen? What were the exact circumstances?
    - Have you ever found it difficult to (again, ask for specific stories)
      - …get questions about your medications answered?
      - …communicate with your doctor about changes in your prescriptions?
      - …get your medications from the pharmacy without having to wait too long?
      - …pay your co-pay?
    - Can you recall a time when you didn’t take you medication for several days?
      - What happened that prevented you from taking your medications?

1. What are some of the other things going on in your life that make it hard for you to take your medications?

Probes:

- - - Do you ever have trouble remembering to take your medications? Why/Why not?
    - Do you ever have trouble remembering to refill your medications? Why/Why not?
    - Do you experience any side effects that make you not want to take your medications?

1. What are the things the VA currently does that make it easier to take your medications?

Probes:

- - - Are there things the pharmacy does that make it easier for you to order or pick up your medications?
    - Are there things that the pharmacy or your doctor does to help you understand the medications you are taking?
    - Are there any other services you’ve encountered at the VA that have helped you take you medication?

1. Are you aware of any programs at the VA that are designed to help you manage your diabetes? What are they and what have you heard?

Probes:

- - - Have you actually participated in any programs?
    - What did the program do that helped you take your medications?
    - How could this program be more helpful to you? What changes to the program would you recommend?
    - What, if anything, did the program you participated in do to help you manage your diabetes?

1. What other things could the VA do to help you take your medications?
2. What are the 2 changes the VA could make that would most help you take your medications?
3. What are the 2 changes you could make in your own life that would most help you take your medications?
